# Induction of STAT1 Phosphorylation at Serine 727 and Expression of Proinflammatory Cytokines by Porcine Reproductive and Respiratory Syndrome Virus

**DOI:** 10.1371/journal.pone.0061967

**Published:** 2013-04-24

**Authors:** Ying Yu, Rong Wang, Yuchen Nan, Linsheng Zhang, Yanjin Zhang

**Affiliations:** 1 College of Life Sciences, Northwest A&F University, Yangling, Shaanxi, China; 2 Molecular Virology Laboratory, VA-MD Regional College of Veterinary Medicine and Maryland Pathogen Research Institute, University of Maryland, College Park, Maryland, United States of America; University of Kansas Medical Center, United States of America

## Abstract

Porcine reproductive and respiratory syndrome virus (PRRSV) is a viral pathogen that causes acute respiratory illnesses in young pigs. Since 1987, PRRSV has contributed substantial economic losses to the swine industry. Elevation of proinflammatory cytokines in PRRSV-infected pigs is thought to contribute to PRRSV pathogenesis. In this study, PRRSV VR-2385, a Type 2 strain with moderate virulence, was found to induce phosphorylation of signal transducer and activator of transcription 1 (STAT1) at serine 727 (pSTAT1-S727) in MARC-145 cells. No phosphorylated STAT1 at tyrosine 701 was detected, which indicates that the pSTAT1-S727 elevation was interferon-independent. The PRRSV-induced pSTAT1-S727, however, was dose-dependent and its levels increased with infection time. IngelVac PRRS MLV strain had a minimal effect on pSTAT1-S727. Compared to MLV-infected cells, VR-2385 infection caused significantly higher level of expression of proinflammatory cytokines, including interleukin 1 beta (IL-1beta) and IL-8. The VR-2385-induced pSTAT1-S727 and cytokine expression were reduced after SB203580, an inhibitor of p38 mitogen-activated protein kinase (MAPK), or methylthioadenosine (MTA), a methyl transferase inhibitor, was added to the cells. The SB203580 and MTA-mediated inhibition suggested that the virus-induced pSTAT1-S727 was dependent on p38 MAPK pathway. In primary porcine alveolar macrophages (PAMs), VR-2385 also induced pSTAT1-S727 and expression of proinflammatory cytokines and chemokines, including IL-1beta, IL-8, chemokine ligand 2 (CCL2) and chemokine (C-X-C motif) ligand 10 (CXCL10). Similarly, SB203580 treatment of PAM cells blocked the elevation of pSTAT1-S727 and cytokine expression. Overexpression of individual viral proteins showed that non-structural protein 12 (nsp12) was able to induce elevation of pSTAT1-S727 and the expression of IL-1β and IL-8. These results indicated that PRRSV VR-2385 induces pSTAT1-S727 and the expression of proinflammatory cytokines, which contributes to the insight of PRRSV pathogenesis.

## Introduction

Porcine reproductive and respiratory syndrome (PRRS) is a widespread viral disease that has caused substantial economic losses to the swine industry [1]. The disease remains a major challenge since it was first reported in the United States in 1987. Moreover, outbreaks of highly pathogenic PRRS in Asia in recent years [2], [3] raise further concerns. The causative agent of PRRS is the PRRS virus (PRRSV), an enveloped, positive-sense, single-strand RNA virus belonging to the genus *Arterivirus*, family *Arteriviridae*, order *Nidovirales* [4]. The entire genome of PRRSV is approximately 15 kb including ten open reading frames (ORFs). The ORF1a and ORF1b encode pp1a and pp1ab polyproteins, which are cleaved into fourteen non-structural proteins [5], [6]. In addition to the two large ORFs, PRRSV genome contains the other eight ORFs: 2, 2a, 3, 4, 5, 5a, 6 and 7 that encode eight structural proteins: GP2, E, GP3, GP4, GP5, 5a, M and N, respectively [7]–[10].

PRRSV propagation *in vitro* relies on an epithelial-derived monkey kidney cell line, MARC-145 [11], and primary cultures of porcine pulmonary alveolar macrophages (PAMs). PAMs are the primary target cells for PRRSV during its acute infection in pigs [12]. Many efforts to control PRRS, including attenuated live virus vaccines, have been tested, but few are successful because of the antigenic and genomic diversity among PRRSV isolates, as well as the persistence of the virus in infected herds.

PRRSV causes acute phase response in pigs by replicating in the lungs and lymphoid organs. Up-regulated proinflammatory cytokines, such as interleukin 1 (IL-1), IL-6 and tumor necrosis factor alpha (TNF-α), initiate this acute phase response and relate to intrinsic pathogenicity in the respiratory infection [13]–[15]. Expression of these cytokines correlates with the severity of pulmonary pathology and the number of macrophages in lung lesion [16]. Evaluation of early cytokine responses to PRRSV infection showed that three serum cytokines IL-8, IL-1β, and IFN-γ were correlated with virus level in pigs [17]. These studies have shown the importance of proinflammatory cytokines in PRRSV infection, but the induction of these genes in PRRSV infection is not well-defined.

In this study, a moderate virulent strain VR-2385 was found to induce phosphorylation of STAT1 (signal transducer and activator of transcription 1) at serine 727 (pSTAT1-S727) in MARC-145 and PAM cells. The virus infection increased expression of some proinflammatory cytokines, including IL-1β and IL-8. Inhibition of p38 mitogen-activated protein kinase (MAPK) blocked elevation of pSTAT1-S727 and expression of the cytokine genes in VR-2385-infected cells. Overexpression of individual viral proteins showed that nsp12 was possibly responsible for the upregulation of pSTAT1-S727.

## Results

### PRRSV Infection of MARC-145 Cells Induces Phosphorylation of STAT1 Serine 727

**Figure 1:**
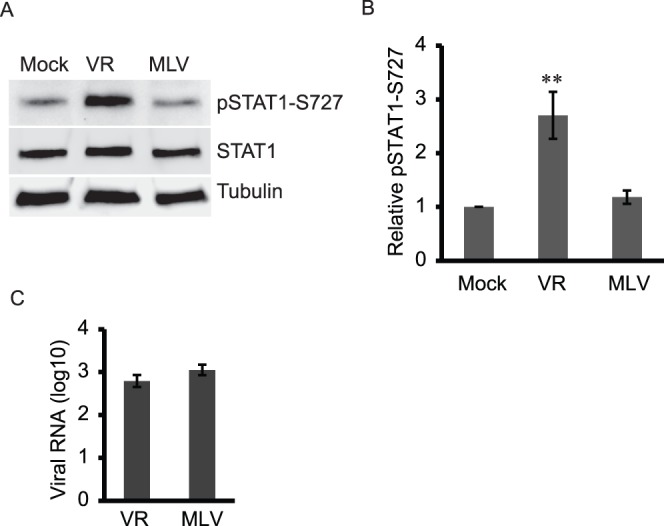
PRRSV VR-2385 induces elevation of phosphorylated STAT1 at serine 727 (pSTAT1-S727) in MARC-145 cells. **A.** VR-2385 (VR) induces higher level of pSTAT1-S727 in MARC-145 cells than MLV. The infected cells were harvested at 24 hpi for Western blotting with antibodies against pSTAT1-S727, STAT1 and tubulin. Lysate of mock-infected cells was included as a control. **B.** Densitometry analysis of pSTAT1-S727 bands to show relative level of pSTAT1 in comparison with mock-infected cells as folds after normalization with tubulin. Significant difference from mock-infected control is shown by “**”, which indicates *P*<0.01. Errors bars indicate standard errors of repeated experiments. **C.** PRRSV viral RNA copies in the PRRSV-infected cells detected by real-time RT-qPCR and shown as log10 per 10 ng total RNA.

During our study of PRRSV inhibition of interferon-activated signaling, we noticed that PRRSV VR-2385 induced the elevation of phosphorylated STAT1 at serine 727. STAT1 is an essential transcription factor for the expression of the majority of IFN-induced genes [18], [19]. MARC-145 cells were inoculated with two different Type 2 PRRSV strains, VR-2385, a moderate virulent strain, and MLV, an avirulent strain, both at 1 multiplicity of infection (MOI). Mock-infected cells were included as controls. The level of pSTAT1-S727 in VR-2385-infected cells 24 h post infection (hpi) was considerably increased in comparison to the mock-infected cells (Fig. 1A). The MLV infection had a minimal effect on pSTAT1-S727 level. Densitometry analysis showed that the pSTAT1-S727 level in VR-2385-infected cells was 2.7-fold higher than that of the mock-infected cells (Fig. 1B). It has been proven that the phosphorylation on both tyrosine 701 and serine 727 residues is required for the interferon-activation of STAT1 [20]. The STAT1 phosphorylation at tyrosine 701 in either PRRSV-infected or mock-infected MARC-145 cells was below detection level (result not shown), which indicates that interferon activated signal transduction was not involved in the pSTAT-S727 in VR-2385-infected cells. The total STAT1 levels in the virus-infected cells were similar to that in mock-infected cells. The results show that VR-2385 induced the elevation of pSTAT1-S727 level in an interferon-independent manner, whereas MLV had a minimal effect on the pSTAT1.

RT-qPCR showed that viral RNA copies were 2.8 and 3.0 log10 per 10 ng total RNA for VR-2385 and MLV-infected cells, respectively (Fig. 1C). This indicates that both VR-2385 and MLV had similar viral load in MARC-145 cells.

### VR-2385 Induces pSTAT1-S727 Elevation in a Dose-dependent and Time-dependent Manner

**Figure 2:**
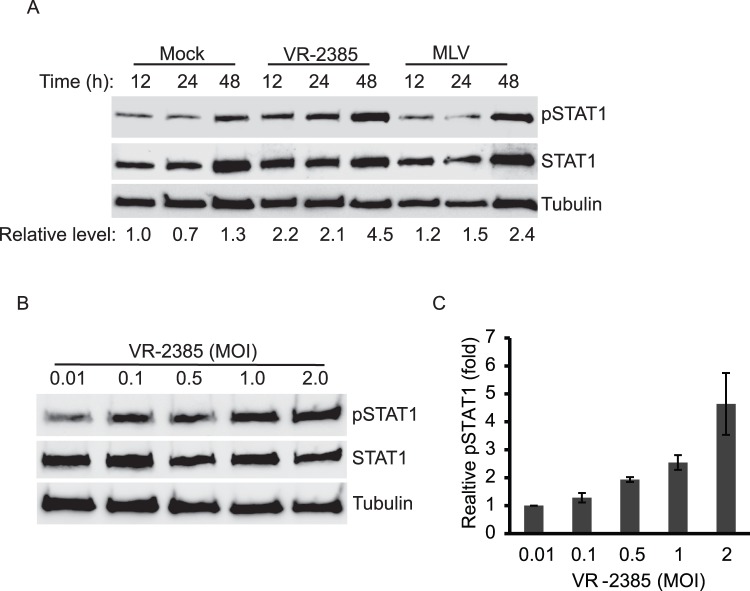
Kinetics and dose-dependent effect of PRRSV-induced elevation of pSTAT1-S727 (pSTAT1). **A.** Kinetics of pSTAT1-S727 in VR-2385-infected cells. MARC-145 cells were infected with VR-2385 and MLV. The cells were harvested at 12, 24 and 48 hpi for Western blotting. Relative levels of pSTAT1 in comparison to the mock-infected cells of 12 hpi time point are shown as folds below the images. **B.** The pSTAT1 level increases along with incremental addition of VR-2385 virus inoculum. The MARC-145 cells were infected with 0.01, 0.1, 0.5, 1.0 and 2.0 MOI and harvested at 24 hpi for Western blotting. **C.** Densitometry analysis of pSTAT1 showing relative levels of pSTAT1 in comparison with cells inoculated with 0.01 MOI as folds after normalization with tubulin. Error bars indicate standard errors among three repeated experiments.

To further examine the induction of pSTAT1-S727 by VR-2385 virus, we conducted a time-kinetic study to determine pSTAT1-S727 levels in MARC-145 cells at different points of time. MARC-145 cells were infected with VR-2385 or MLV at 1 MOI and were harvested at 12, 24, and 48 hpi for Western blotting assay. Compared to mock-infected cells at 12 hpi, the levels of pSTAT1-S727 in VR-2385-infected cells at 12, 24, and 48 hpi were 2.2, 2.1 and 4.5-fold, respectively (Fig. 2A). These results demonstrate that VR-2385 induced elevation of pSTAT1-S727 in a time-dependent manner. The levels of pSTAT1-S727 in MLV-infected cells at 12, 24, and 48 hpi were 1.2, 1.5 and 2.4-fold, respectively, in comparison with the mock-infected cells. The pSTAT1-S727 level in mock-infected cells changed minimally at each time point. Levels of total STAT1 and tubulin increased in the mock- or MLV-infected cells at 48 hpi, which might account for the elevation of pSTAT1-S727 levels in these cells. The increase of the total proteins could be the result of cell proliferation.

Next we tested whether the pSTAT1-S727 level would increase with incremental VR-2385 inoculum. MARC-145 cells were inoculated with VR-2385 at different MOI and harvested at 24 hpi for Western blotting. The pSTAT1-S727 level increased along with incremental MOI of VR-2385 (Fig. 2B). The levels of pSTAT1-S727 in the cells infected with 0.01, 0.1, 0.5, 1.0 and 2.0 MOI were 1.0, 1.3, 1.9, 2.5 and 4.6-fold, respectively (Fig. 2C). The results showed that pSTAT1-S727 level increased with incremental VR-2385 MOI, while total STAT1 level showed minimal change. The dose-dependent effect confirmed that the elevation of pSTAT1-S727 level was due to VR-2385 infection.

### The PRRSV-induced pSTAT1-S727 is Correlated with p38 MAPK Signaling Pathway

**Figure 3:**
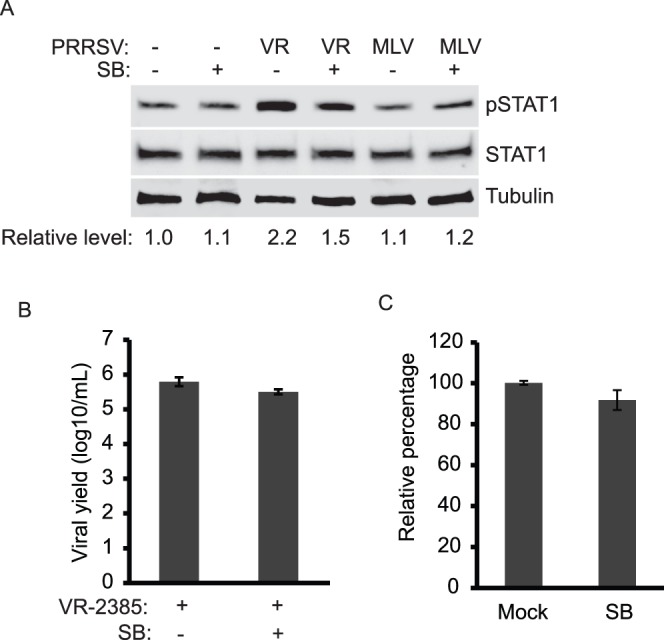
The p38 MARK signaling pathway is involved in pSTAT1-S727 elevation in PRRSV-infected cells. **A.** SB203580 treatment leads to inhibition of PRRSV-induced pSTAT1 elevation. MARC-145 cells were infected with PRRSV and treated with SB203580. At 24 hpi, the cells were harvested for Western blotting with antibodies against pSTAT1-S727, STAT1, and tubulin. **B.** PRRSV yield remains unchanged in the presence of SB203580. Cell culture supernatant samples of PRRSV-infected MARC-145 cells in the presence or absence of SB203580 were titrated. The viral yield is shown as log10 TCID50/ml. **C.** Cell viability assay of SB203580-treated MARC-145 cells. The cells were subjected for the assay 24 h after treatment. Relative percentages in comparison with mock-treated cells are shown.

It is proven that p38 MAPK is required for the stress-induced STAT1 phosphorylation at serine 727 [21]. SB203580, a specific inhibitor of p38 MAPK [22], was used to test whether VR-2385-induced elevation of pSTAT1-S727 depends on p38 MAPK. In this test, MARC-145 cells were treated with SB203580 at a final concentration of 20 µM after incubation with VR-2385 or MLV inoculum. Compared to mock-treatment, SB203580 treatment of VR-2385-infected cells led to a considerable reduction of pSTAT1-S727 (Fig. 3A). The levels of pSTAT1-S727 in VR-2385-infected cells in the presence or absence of SB203580 treatment were 1.5 and 2.2-fold, respectively, in comparison with mock-infected cells. For the mock-infected or MLV-infected cells, SB203580 treatment led to minimal change of pSTAT1-S727. The total STAT1 level remained stable in the cells treated with SB203580.

To exclude the possibility that the SB203580 treatment had any adverse effect on VR-2385 replication in MARC-145 cells, viral yields in the cell culture supernatant samples were titrated. The viral titers were 5.5 log10 TCID_50_/ml in the presence of SB203580 and 5.8 in the absence of the chemical (Fig. 3B). These results demonstrate that SB203580 treatment of the cells had a minimal effect on VR-2385 replication. In addition, to ensure that the SB203580 treatment had no adverse effect on MARC-145 cells, cell viability assay was performed. SB203580-treated MARC-145 cells had 92% cell viability (Fig. 3C), which confirms that the SB203580 treatment had minimal adverse effect on cell viability.

Collectively, these results show that p38 MAPK was involved in VR-2385-induced elevation of pSTAT1-S727 in MARC-145 cells.

### Elevated Expression of Proinflammatory Cytokine Genes in PRRSV-infected Cells is Correlated with Increased pSTAT1-S727 Level

**Figure 4:**
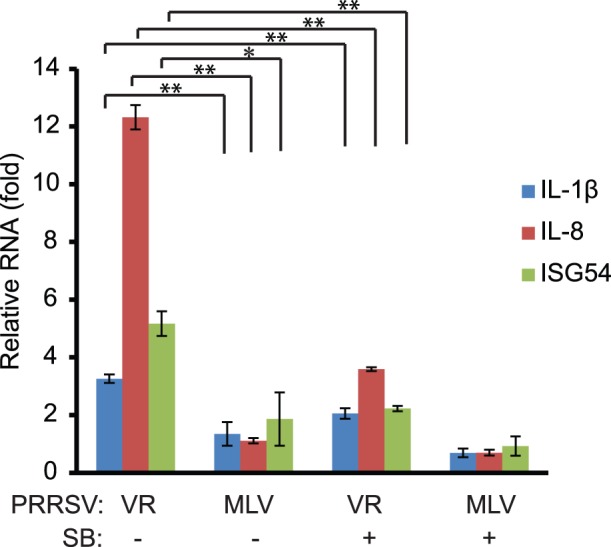
VR-2385 infection leads to higher expression of proinflammatory cytokine genes than MLV and SB203580 treatment reduces their expression. MARC-145 cells were infected with VR-2385 or MLV and treated with SB203580. Mock-treated cells were included for control. The cells were harvested at 48 hpi for RNA isolation and RT-qPCR. Relative folds of transcript levels of IL-1β, IL-8, and ISG54 in comparison with mock-treated PRRSV-negative cells are shown. Error bars represent variation of three repeated experiments. Significant differences between paired samples are shown by “*” and “**”, which indicate *P*<0.05 and *P*<0.01, respectively.

As STAT1 is involved in activating the expression of many cellular genes [23], we wondered whether VR-2385 causes the elevation of pSTAT1-S727 which can, in turn, lead to the up-regulated expression of proinflammatory cytokines. First, MARC-145 cells were infected with VR-2385 or MLV, and harvested for RNA isolation. RT-qPCR was performed to detect transcripts of the following cellular genes: IL-1β, IL-6, IL-8, IL-10, IL-12, interferon regulatory factor 1 (IRF-1), IRF-7, TNF-α, interferon stimulated gene 15 (ISG15), ISG54, ISG56, chemokine (C-X-C motif ) ligand 10 (CXCL10), and chemokine ligand 2 (CCL2, also known as monocyte chemotactic protein*-*1 (MCP-1)). Of the genes tested, the transcripts of IL-1β, IL-8 and ISG54 increased significantly in VR-2385-infected cells by 3.3, 12.3 and 5.2-fold (Fig. 4). MLV-infection caused minimal change in the expression of these three genes. The levels of the three transcripts induced by VR-2385 were significantly higher than those in MLV-infected cells.

SB203580 treatment of VR-2385-infected cells led to a significant reduction of the transcripts of IL-1β, IL-8 and ISG54 to 2.1, 3.6 and 2.2-fold (Fig. 4). The results suggest that VR-2385 induction of pSTAT1-S727 caused increased expression of these proinflammatory cytokine genes and that p38 MAPK was required in the activation of pSTAT1. In contrast, MLV infection did not induce the expression of these three cytokine genes, which is consistent with the inability of MLV to induce pSTAT1-S727.

### PRRSV-induced pSTAT1-S727 and Expression of Proinflammatory Cytokine Genes are Sensitive to Methyl Transferase Inhibitor

**Figure 5:**
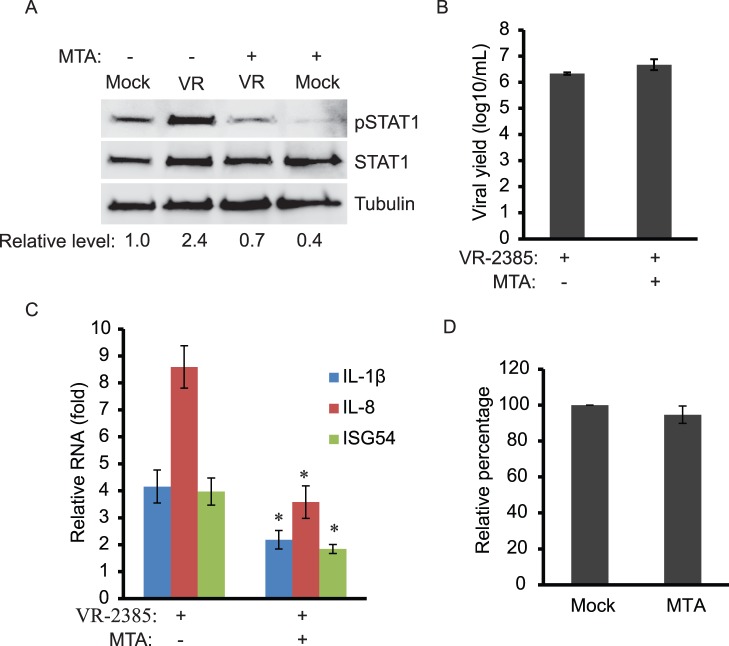
Inhibition of PRRSV-mediated STAT1 phosphorylation by methylthioadenosine (MTA). **A.** MTA treatment leads to blockage of VR-2385-induced STAT1 phosphorylation. MARC-145 cells were infected with VR-2385 and treated with MTA. The cells were harvested at 24 hpi for Western blotting with antibodies against pSTAT1-S727, STAT1, and tubulin. Relative levels of pSTAT1 in comparison to the mock-treated and uninfected cells are shown as folds below the images. **B.** PRRSV yield remains stable in the presence of MTA. The virus samples were titrated and the result is shown as log10 TCID50/ml. **C.** MTA treatment reduces PRRSV-induced expression of proinflammatory cytokine genes. MARC-145 cells were infected with VR-2385 and treated with MTA. The cells were harvested at 48 hpi for RNA isolation and RT-qPCR. “*” indicates significant differences (*P*<0.05) between MTA-treated and mock-treated cells. **D.** Cell viability assay of MTA-treated MARC-145 cells. The cells were subjected for the assay 24 h after treatment. Relative percentages in comparison with mock-treated cells are shown.

In order to confirm that p38 MAPK contributed to the VR-2385-induced pSTAT1-S727 elevation, we used another inhibitor, methylthioadenosine (MTA), a potent inhibitor of carboxyl-methyl transferase, which has been shown to inhibit p38 MARK [24] and STAT1 methylation and activation [25]. MARC-145 cells were treated with MTA at a final concentration of 1 mM after VR-2385 infection. Western blotting showed that compared to mock-infected cells, the level of pSTAT1-S727 in the VR-2385-infected cells was increased to 2.4-fold, but dropped to 0.7-fold in the presence of MTA (Fig. 5A). Total STAT1 levels showed minimal change. Cell culture supernatant samples were titrated for viral yield. The virus-infected cells in the presence of MTA treatment had viral yield similar to the infected cells in the absence of MTA (Fig. 5B).

RT-qPCR analysis showed that MTA treatment led to a significant reduction of VR-2385-mediated expression of IL-1β, IL-8 and ISG54 from 4.2, 8.6 and 4.0-fold to 2.2, 3.6 and 1.8-fold (Fig. 5C). The results were consistent with SB203580 treatment and confirmed that VR-2385-induced elevation of pSTAT1-S727 is dependent on p38 MAPK. In addition, cell viability assay was performed to make sure that the MTA treatment had no adverse effect on the cells. Compared to the mock-treated control, MTA-treated MARC-145 cells had 95% cell viability (Fig. 5D). This proves that the MTA treatment in this experiment had minimal adverse effect on growth of MARC-145 cells.

### VR-2385 Infection of Primary Porcine Pulmonary Alveolar Macrophages also Induces Elevation of pSTAT1-S727

**Figure 6:**
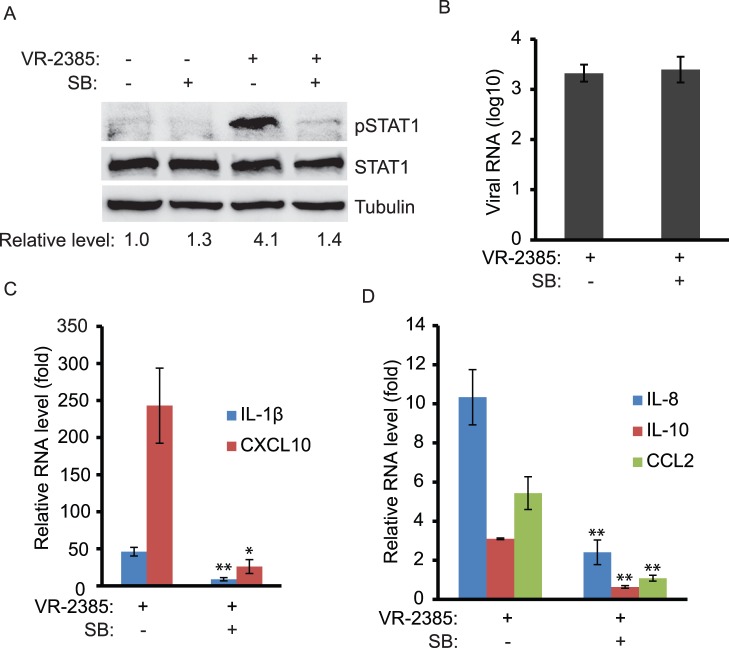
PRRSV infection of PAM cells leads to elevation of pSTAT1-S727. **A.** Elevation of pSTAT1-S727 in VR-2385-infected PAM cells detected by Western blotting. The cells were infected with VR-2385 and treated with SB203580. Mock-infected and mock-treated cells were included as controls. The blotting was done with antibodies against pSTAT1-S727, STAT1 and tubulin. Relative levels of pSTAT1-S727 compared to the mock-treated control are shown as folds below the images. **B.** PRRSV viral RNA copies in PAMs detected by RT-qPCR and shown as log10 per 10 ng total RNA. **C&D.** Increased expression of IL-1β, CXCL10, IL-8, IL-10, and CCL2 in PRRSV-infected PAMs detected by RT-qPCR. The cells were infected with VR-2385 and treated with SB203580. Relative levels of gene expression are shown as folds in comparison with the mock-treated PRRSV-negative cells.

As PAMs are the primary target cells of PRRSV *in vivo*, we tested whether VR-2385 would induce pSTAT1-S727 elevation in the cells. PAMs were infected with VR-2385 with 0.05 MOI and were treated with SB203580 after virus inoculation. Mock-treated and mock-infected cells were included as a control. The cells were harvested for Western blotting at 12 hpi. Compared to mock-infected cells, VR-2385 infection led to a significant elevation of pSTAT1-S727 by 4.1-fold (Fig. 6A). The addition of SB203580 to the virus-infected cells blocked the elevation of pSTAT1-S727. RT-qPCR showed that the viral RNA copies in the VR-2385 infected cells were 3.3 log10 in the presence of SB203580 treatment and 3.4 log10 in the absence of the chemical, indicating that the inhibitor had no detectable adverse effect on VR-2385 viral RNA load (Fig. 6B). These results were consistent with those from MARC-145 cells.

RT-qPCR results showed that the expression of five cytokine or chemokine genes was significantly increased by VR-2385 infection. In addition to IL-1β and IL-8, IL-10, CXCL10 and CCL2 were also up-regulated. The transcripts of IL-1β and CXCL10 in VR-2385-infected cells were increased to 46 and 243-fold, respectively (Fig. 6C). SB203580 treatment of the virus-infected cells reduced the transcripts of IL-1β and CXCL10 to 8.9 and 26-fold, respectively. The transcripts of IL-8, IL-10 and CCL2 in the virus-infected PAMs were increased to 10.3, 3.1 and 5.4-fold, respectively (Fig. 6D). The levels of the IL-8, IL-10 and CCL2 transcripts in the virus-infected cells after SB203580 treatment were reduced to 2.4, 0.6 and 1.0-fold, respectively. This demonstrates that VR-2385 infection caused the elevation of pSTAT1-S727 and expression of the cytokine and chemokine genes in PAM cells.

### Screening of Viral Proteins that May Correlate with the Elevation of pSTAT1-S727

**Figure 7:**
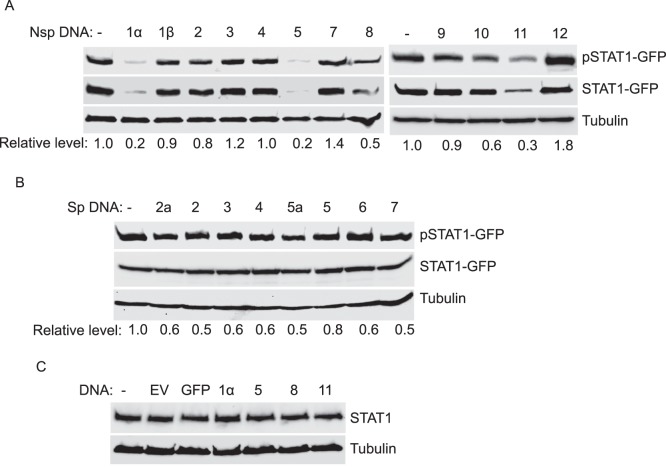
Screening of non-structural and structural proteins of PRRSV VR-2385 in inducing pSTAT1-S727 elevation. **A.** Screening of VR-2385 non-structural proteins (nsps) 1–12. HEK293 cells were transfected with STAT1-GFP and VR-2385 nsp plasmids. The cells were harvested 48 h after transfection for Western blotting with antibodies against pSTAT1-S727, STAT1 and tubulin. Relative levels of pSTAT1 in comparison with control vector lane are shown as folds below the images. **B.** Screening of VR-2385 structural proteins (sps) encoded by ORFs 2–7. HEK293 cells were transfected with STAT1-GFP and VR-2385 sp plasmids. Blotting and analysis were conducted similarly as in “A”. **C**. Expression of endogenous STAT1 remains stable in HEK293 cells transfected with PRRSV nsps. The cells were transfected with empty vector, GFP, nsp1α, nsp5, nsp8 or nsp11 plasmids. Western blotting with antibodies against STAT1 and tubulin was conducted.

To identify which viral proteins of VR-2385 could correlate with the induction of pSTAT1-S727, we constructed a series of pCAGEN plasmids containing structural and non-structural viral genes. As the endogenous pSTAT1-S727 in HEK293 cells was below detection level, overexpression of STAT1-GFP was used in this assay. HEK293 cells were co-transfected with STAT1-GFP and the viral plasmids. At 48 h after the transfection, the cells were collected for Western blotting assay. The cells co-transfected with STAT1-GFP and pCAGEN-GFP were included for control. Among the nsp plasmids, only nsp12 induced considerable elevation of pSTAT1-S727, which was 1.8-fold in comparison with the vector control (Fig. 7A). None of the structural proteins led to significant increase of pSTAT1-S727 (Fig. 7B). Interestingly, a few viral nsps, including nsp1α, 5, 8 and 11, led to a reduction of total STAT1-GFP level. To eliminate the possibility that these nsps had any adverse effect on endogenous STAT1 expression, we transfected HEK293 cells with empty vector, GFP and the four nsp plasmids not including STAT1-GFP. Total STAT1 levels showed minimal change in the cells transfected with either GFP or the four nsp plasmids (Fig. 7C). The results indicate that the four nsps did not have adverse effect on endogenous STAT1 expression, however, they did exert a negative effect on expression of exogenous STAT1-GFP. According to our results, nsp12 is likely the viral protein able to induce pSTAT1-S727 elevation.

### Nsp12 Induces the Elevation of pSTAT1-S727 and Expression of the Proinflammatory Cytokines

**Figure 8:**
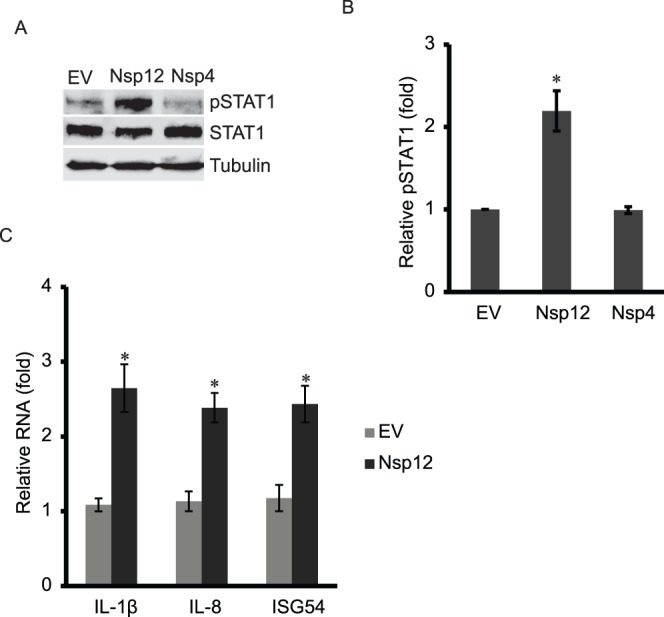
Nsp12 induces elevation of pSTAT1-S727 and expression of proinflammatory genes in HEK293 cells. **A.** Nsp12 induces elevation of pSTAT1-S727 in HEK293 cells. The cells were transfected with nsp12 or nps4 plasmids. The cells were harvested 48 h after transfection for Western blotting with antibodies against pSTAT1-S727, STAT1 and tubulin. **B.** Densitometry analysis showing relative pSTAT1 levels in comparison with empty vector lane after normalization with tubulin. **C.** Nsp12 induces expression of proinflammatory cytokine genes. HEK293 cells were transfected with nsp12 plasmid and harvested for RT-qPCR. Significant differences between cells transfected with nsp12 and those with empty vector are shown by “*”, which indicates *P*<0.05.

To verify that nsp12 was the viral protein inducing pSTAT1-S727, we examined endogenous pSTAT1-S727 in HEK293 cells with nsp12 overexpression by loading 12-fold more total proteins in SDS-PAGE than the test above. Nsp4, which did not lead to elevation of pSTAT1-S727 in the cells with overexpression of STAT1-GFP, was included as a control. Compared to the empty vector control, the cells with nsp12 expression showed considerably higher level of pSTAT1-S727 (Fig. 8A). Nsp4 has a minimal effect on pSTAT1-S727 level. The total STAT1 levels exhibited minimal change. Densitometry analysis showed that nsp12 expression led to elevation of pSTAT1-S727 by 2.2-fold in comparison with the empty vector control (Fig. 8B).

RT-qPCR showed that in HEK293 cells with nsp12 expression, the transcripts of IL-1β, IL-8 and ISG54 increased 2.6, 2.4 and 2.4-fold, respectively, compared to the cells with the empty vector control (Fig. 8C). The results indicate that nsp12 induced elevation of pSTAT1-S727 as well as the expression of the proinflammatory cytokine genes.

## Discussion

STAT1 is a protein belonging to the STAT family and a key player in response to extracellular stimuli to activate transcription of downstream genes. PRRSV inhibits interferon-activated JAK-STAT pathway [26], yet it induces upregulation of proinflammatory cytokines in pigs. The question is how the virus can activate the expression of the cytokine genes while blocking JAK-STAT pathway. This study offers the possibility that PRRSV induces expression of the proinflammatory cytokines via activation of STAT1 serine 727 phosphorylation and the pSTAT1 activation depends on p38 MAPK pathway in an interferon-independent manner. The conclusion was drawn through several experiments in PRRSV-infected MARC-145 cells and confirmed in primary PAMs. Moderate virulent strain VR-2385 induced pSTAT1-S727, while avirulent MLV did not. The STAT1 tyrosine 701 phosphorylation could not be detected, which indicates that, as expected, IFN-induced JAK-STAT pathway was not activated. Thus, the pSTAT1-S727 elevation in VR-2385-infected cells was not interferon-dependent. MLV had similar viral RNA load as VR-2385, which excludes the possibility that the minimal change of pSTAT1-S727 in MLV-infected cells was the result of low MLV replication.

The pSTAT1-S727 levels increased with extension of VR-2385 infection from 12 to 48 hpi. The continuous presence of pSTAT1-S727 is different from interferon-induced transient phosphorylation of STAT1 tyroine-701 in PRRSV-infected cells, shown in an earlier publication [26]. In MLV-infected cells, the pSTAT1-S727 also increased at 48 hpi, the late stage for virus replication. The reason for the increase might be that MLV replication induces cytopathic effect at the late stage, which stressed the cells. Also, the whole STAT1 and tubulin levels in MLV-infected cells increased. The increase of the total proteins could be a result of cell proliferation. The VR-2385 infection induced pSTAT1-S727 elevation in a dose-dependent manner, which verified the specific effect due to VR-2385 virus replication.

It is proven that p38 MAPK pathway is required for pSTAT1-S727 induced by lipopolysaccharide (LPS) or ultraviolet (UV) light [21], [27]. VR-2385 induced pSTAT1-S727 elevation was found to be dependent on p38 MAPK pathway too as the phosphorylation level was reduced by treatment with inhibitor SB203580. The p38-specific inhibitor caused considerable reduction of pSTAT1-S727 in VR-2385-infected cells but had no effect on basal pSTAT1-S727 in MLV-infected or mock-infected cells. The SB203580 treatment had minimal adverse effects on cell growth and virus propagation, which excluded the possibility of cytotoxicity.

Of the cellular genes screened, IL-1β, IL-8 and ISG54 were significantly up-regulated by VR-2385 infection of MARC-145 cells. SB203580 treatment blocked the expression of these genes in the cells with VR-2385 replication. The results indicate that VR-2385 induced the expression of these genes and that p38 MAPK pathway was involved. The up-regulated expression of IL-1β in VR-2385-infected cells is consistent with a previous report showing the elevation of IL-1 in PRRSV-infected pigs [13]. These cytokines may correlate with PRRSV pathogenesis because of their proven functions narrated below. IL-1 is able to enhance the expression of the adhesion molecules which are essential for pulmonary inflammation. IL-8, a chemoattractant cytokine, is a major factor attracting neutrophils to the lung [28]. ISG54 is an antiviral protein induced by viral infection and interferons. The ISG54 gene codes for a protein of ∼54 kDa (472 aa) with tetratricopeptide repeats, which is also known as interferon-induced protein with tetratricopeptide repeats 2 (IFIT2). It is one of the four IFITs with the characteristic repeats. ISG54 was also found to induce apoptosis [29].

The VR-2385-induced pSTAT1-S727 elevation was also verified with MTA, an inhibitor of methyl transferase. The inhibitor blocked elevation of the virus-induced pSTAT1-S727. MTA is reported to inhibit multiple pathways including ERK, p38 MAPK pathway and STAT1 methylation [24], [25], [30]. The result further substantiated the p38 MAPK involvement in the virus-induced pSTAT1-S727. MTA treatment also caused the reduction of the basal level of pSTAT1-S727 in mock-infected cells. This indicates that the inhibitor worked on multiple pathways that affect the basal level of pSTAT1-S727. After MTA treatment, the expression of IL-1β, IL-8 and ISG54 in VR-2385-infected cells was significantly reduced, which was consistent with the reduction of pSTAT1-S727.

The VR-2385-induced pSTAT1-S727 was also verified in primary PAM cells. SB203580 treatment blocked the elevation of pSTAT1-S727. Similar to MARC-145 cells, the virus infection resulted in increased expression of IL-1β, IL-8, IL-10, CCL2 and CXCL10 in PAMs. The transcripts of the later three genes did not change in PRRSV-infected MARC-145 cells, which might be related to cell type differences. The elevation of CCL2 and CXCL10 is consistent with our previous data [31] and the macrophage infiltration in the lungs of PRRSV-infected pigs [32]. IL-10 is capable of inhibiting the production of pro-inflammatory cytokines in macrophages, such as TNF-α, IL-6, and IL-12 [33]. This is consistent with our results showing that there was no change in expression of these three genes in VR-2385-infected PAMs. The upregulation of IL-10 is consistent with a previous report that showed induction of IL-10 depending on p38 MAPK in PRRSV-infected macrophages [34], [35]. CCL2 and CXCL10 are chemokines that are low molecular weight molecules and play a pivotal role in the orchestration of an effective antiviral immune response, partly by attracting leukocytes to the site of inflammation or infection [36]. Modulating expression of chemokine and proinflammatory chemokine genes during PRRSV infection might correlate with PRRSV pathogenesis.

Viral proteins of VR-2385 were also screened as potential contributors to the increase of pSTAT1-S727. The nsp12 was found to induce pSTAT1-S727 in HEK293 cells. Furthermore, the expression of the proinflammatory cytokine genes was also up-regulated. The nsp12 has 153 amino acids with predicted molecular weight of 17 kDa with unknown functions. Our data suggests that, by modulating cellular gene expression, nsp12 might be involved in PRRSV pathogenesis. More studies are needed to identify the precise functions of nsp12 in PRRSV infection.

In conclusion, PRRSV VR-2385 induces pSTAT1-S727 elevation via p38 MAPK pathway. The STAT1 activation correlates with the expression of the proinflammatory cytokine genes. Of the viral proteins, nsp12 is the protein that contributes to the induction of pSTAT1-S727. These results contribute to insights of PRRSV pathogenesis by showing the activation of pSTAT1 in induction of proinflammatory cytokines.

## Materials and Methods

### Cells and Viruses

MARC-145 [11] and HEK293 (ATCC, CRL-1573) cells were maintained at 37°C in Dulbecco’s modified Eagle’s minimal essential medium (DMEM) supplemented with 10% fetal bovine serum (FBS) containing 100 units/ml penicillin and 0.1 mg/ml streptomycin at 37°C and 5% CO_2_. PAM cells were prepared from bronchoalveloar lavage from 4-week-old pigs free of PRRSV, as previously described [37]. The PAM cells were maintained in RPMI 1640 supplemented with 10% FBS. PRRSV strains VR-2385 and MLV were propagated and titrated as described previously [38]. Chemical SB203580 and MTA (Sigma, St. Louis, MO) were used at a final concentration of 20 µM and 1 mM, respectively, to treat cells for the length of time as indicated.

### RNA Extraction, Reverse Transcription and Real-time PCR (RT-qPCR)

Total RNA of PAM and MARC-145 cells were extracted with Trizol (Life Technologies Corporation, Carlsbad, CA) and RNA*Sound*™ RNA extraction card (FortiusBio, Monterey Park, CA), separately, following the manufacturers’ instructions. For using RNA extraction card, MARC-145 cells were detached from culture plate with trypsin and transferred to a microfuge tube for centrifugation at 400×g for 3 min at 4°C. The cell pellet was resuspended with 20 µl RNase-free water. The cell suspension was added onto 3 mm disc of RNA extraction card. The card was dried at 60°C for 15 min. RNA was eluted with 20 µl RNase-free water from the dry card. First-strand cDNA synthesis and real-time PCR were performed as described previously [26], [31]. Primers used in this study are either published [26], [31], [37] or listed in Table 1. The ribosomal protein L32 (RPL32) transcripts were used as endogenous control for normalization.

**Table 1:** List of primers for real-time PCR.

Name*	Sequences (5′ to 3′)
IL1b-F1	CCCAACTGGTACATCAGCAC
IL1b-R1	GGAAGACACAAATTGCATGG
IL8-F1	AGGACAAGAGCCAGGAAGAA
IL8-R1	ACTGCACCTTCACACAGAGC
ISG54-F1	ATGGAGCAGATTCTGAGGCT
ISG54-R1	GAGGCTTCCAGACTCCAAAC
sIL1b-F1	ACCTGGACCTTGGTTCTCTG
sIL1b-R1	CATCTGCCTGATGCTCTTGT
sIL8-F1	TAGGACCAGAGCCAGGAAGA
sIL8-R1	AGCAGGAAAACTGCCAAGAA
sIL10-F1	CTGCCTCCCACTTTCTCTTG
sIL10-R1	TCAAAGGGGCTCCCTAGTTT

* F1: forward primer, R1: reverse primer. Primers for swine genes start with “s”.

### Western Blotting

Total proteins from cell lysate were separated by sodium dodecyl sulfate-polyacrylamide gel electrophoresis (SDS-PAGE) and transferred onto nitrocellulose membranes, as described [39]. Blotting with antibodies against STAT1 (Santa Cruz Biotechnology, Santa Cruz, CA), pSTAT1-S727 (Catalogue number 07-714, EMD Millipore, Billerica, MA) and β-tubulin (Sigma, St. Louis, MO). The chemiluminescence signals were detected by ChemiDoc XRS imaging system (Bio-Rad Laboratories, Hercules, CA) and intensities of target bands were quantitatively analyzed by using Image Lab Program, Version 4.0 (Bio-Rad Laboratories, Hercules, CA).

### Cell Viability Assay

The effect of SB203580 and MTA on viability of MARC-145 cells was assessed by CellTiter-Glo® Luminescent Cell Viability Assay (Promega, Madison, WI). The cells were treated with SB203580 and MTA for 24 h before analyzed in the assay. The luminescence signal was recorded with VICTOR3™ Multilabel Counter (Perkin-Elmer Life and Analytical Sciences, Wellesley, MA).

### Statistical Analysis

Differences in indicators between treatment groups, such as RNA levels of cellular genes between treated and mock-treated cells, were assessed by a signal factor ANOVA statistical analysis. A two-tailed *P*-value less than 0.05 is considered as statistical significance.
